# Supplementary material for: Sensitivity and specificity of Dried Blood Spot and Plasma Separation Card samples for Hepatitis C Virus RNA Testing
Source: PLOS Glob Public Health. 2026 Mar 11;6(3):e0006082. doi: 10.1371/journal.pgph.0006082 (PMC12978484; doi:10.1371/journal.pgph.0006082)
Supplement: S1 Table — DBS, dried blood spot; PSC, plasma separation card. vDBS, venous dried blood spot; cDBS, capillary dried blood spot; vPSC, venous plasma separation card; cPSC, capillary plasma separation card; Detectable, quantifiable viral load; < titer, detectable, non‑quantifiable, i.e., detectable signal below assay limit of quantification; Undetectable, no viral RNA detected. Indeterminate, invalid/error/no result. (DOCX) [file pgph.0006082.s001.docx]

**S1 Table**. Summary of Repeat Testing Outcomes for Discordant DBS and PSC Samples

| Sample Type | N Re-tested | Previous Result Summary | Repeat Result Summary |
| --- | --- | --- | --- |
| vDBS | 89 | Detected, non-quant or <titer: ~90% (80/89)  Low-positive quantifiable (15–104 IU/mL): ~10% (9/89) | Target Not Detected: ~92% (82/89)  <titer / below LOQ: ~6% (5/89)  Quantifiable VL on repeat (5780 – 10 000 IU/mL): ~2% (2/89) |
| cDBS | 47 | <titer or Detected, non-quant: ~94% (44/47)  Low-positive quantifiable (17–21 IU/mL): ~6% (3/47) | Target Not Detected: ~85% (40/47)  <titer: ~13% (6/47)  Quantifiable VL on repeat (11 000IU/mL): ~2% (1/47) |
| vPSC | 18 | <titer or indeterminate; ~83% (15/18)  Quantifiable (22–16,500 IU/mL): ~17% (3/18) | Target Not Detected: ~56% (10/18)    <titer: ~22% (4/18)  Quantifiable VL on repeat (22 – 178IU/mL): ~22% (4/18) |
| cPSC | 8 | 6: Indeterminate; no repeat testing available as 1 PSC tested per participant)  Quantifiable (49–410,000 IU/mL): 100% (2/2) | No repeat sample available (no sample): 100% (8/8) |

DBS, dried blood spot; PSC, plasma separation card.

vDBS, venous dried blood spot; cDBS, capillary dried blood spot; vPSC, venous plasma separation card; cPSC, capillary plasma separation card;

Detectable, quantifiable viral load;

<titer, detectable, non‑quantifiable i.e. detectable signal below assay limit of quantification;

Undetectable, no viral RNA detected.

Indeterminate, invalid/error/no result
