# Supplementary material for: Sensitivity and specificity of Dried Blood Spot and Plasma Separation Card samples for Hepatitis C Virus RNA Testing
Source: PLOS Glob Public Health. 2026 Mar 11;6(3):e0006082. doi: 10.1371/journal.pgph.0006082 (PMC12978484; doi:10.1371/journal.pgph.0006082)
Supplement: S2 Table — CI, confidence interval; DBS, dried blood spot; PSC, plasma separation card. (DOCX) [file pgph.0006082.s002.docx]

**S2 Table.** Diagnostic accuracy of dried blood spot and Plasma Separation Card samples for detecting hepatitis C virus RNA using the Roche cobas 4800 system, according to site.

|  |  | **Roche cobas 4800 capillary DBS** | |  | **Roche cobas 4800 venous DBS** | |  | **Roche cobas 4800 venous PSC** | |
| --- | --- | --- | --- | --- | --- | --- | --- | --- | --- |
| **Site** | **Reference test and sample type** | **Sensitivity (%),  (95% CI)** | **Specificity (%),  (95% CI)** |  | **Sensitivity (%),  (95% CI)** | **Specificity (%),  (95% CI)** |  | **Sensitivity (%),  (95% CI)** | **Specificity (%),  (95% CI)** |
| All sites | Roche cobas HCV plasma | 97.2 (95.2 – 98.3) | 88.6 (85.4 – 91.2) |  | 96.1 (93.9 – 97.5) | 87.8 (84.5 – 90.4) |  | 95.2 (92.8 – 96.8) | 99.6 (98.5 – 99.9) |
| Georgia | Roche cobas HCV plasma | 99.2 (95.5 – 99.9) | 86.4 (79.9 – 91.0) |  | 99.2 (95.5 – 99.9) | 79.7 (72.5 – 85.4) |  | 98.3 (94.2 – 99.5) | 100 (97.4 – 100) |
| Cameroon | Roche cobas HCV plasma | 97.3 (93.3 – 99.0) | 80.8 (72.0 – 87.4) |  | 97.2 (95.2 – 98.3) | 96.0 (91.5 – 98.2) |  | 95.3 (90.7 – 97.7) | 100 (96.3 – 100) |
| Greece | Roche cobas HCV plasma | 96.7 (90.8 – 98.9) | 90.2 (83.6 – 94.3) |  | 94.6 (87.9 – 97.7) | 90.2 (83.6 – 94.3) |  | 93.5 (86.5 – 97.0) | 100 (96.9 – 100) |
| Rwanda | Roche cobas HCV plasma | 94.6 (88.0 – 97.7) | 97.2 (92.0 – 99.0) |  | 93.5 (86.6 – 97.0) | 100 (96.5 – 100) |  | 92.5 (85.3 – 96.3) | 98.1 (93.4 – 99.5) |

CI, confidence interval; DBS, dried blood spot; PSC, plasma separation card.
