# Supplementary material for: Sensitivity and specificity of Dried Blood Spot and Plasma Separation Card samples for Hepatitis C Virus RNA Testing
Source: PLOS Glob Public Health. 2026 Mar 11;6(3):e0006082. doi: 10.1371/journal.pgph.0006082 (PMC12978484; doi:10.1371/journal.pgph.0006082)
Supplement: S3 Table — CI, confidence interval; DBS, dried blood spot; PSC, plasma separation card. (DOCX) [file pgph.0006082.s003.docx]

**S3 Table**. Diagnostic accuracy of dried blood spot and Plasma Separation Card samples for detecting hepatitis C virus RNA using the Roche cobas 6800 system, according to site.

|  |  | **Roche cobas 6800 venous DBS** | |  | **Roche cobas 6800 capillary PSC** | |  | **Roche cobas 6800 venous PSC** | |
| --- | --- | --- | --- | --- | --- | --- | --- | --- | --- |
| **Site** | **Reference test and sample type** | **Sensitivity (%),  (95% CI)** | **Specificity (%),  (95% CI)** |  | **Sensitivity (%),  (95% CI)** | **Specificity (%),  (95% CI)** |  | **Sensitivity (%),  (95% CI)** | **Specificity (%),  (95% CI)** |
| All sites | Roche cobas HCV plasma | 97.3 (95.4 – 98.5) | 95.9 (93.7 – 97.3) |  | 96.9 (94.8 – 98.1) | 99.8 (98.8 – 100) |  | 96.7 (94.6 – 98) | 99.8 (98.8 – 100) |
| Georgia | Roche cobas HCV plasma | 99.2 (95.5 – 99.9) | 96.6 (92.3 – 98.5) |  | 98.3 (94.2 – 99.5) | 100 (97.5 – 100) |  | 98.4 (94.2 – 99.5) | 100 (97.5 – 100) |
| Cameroon | Roche cobas HCV plasma | 98.6 (95.1 – 99.6) | 97.1 (91.9 – 99.0) |  | 98.6 (95.1 – 99.6) | 99.0 (94.4 – 99.8) |  | 98.6 (95.1 – 99.6) | 100 (96.4 – 100) |
| Greece | Roche cobas HCV plasma | 96.7 (90.8 – 98.9) | 92.7 (86.8 – 96.1) |  | 96.7 (90.8 – 98.9) | 100 (97.0 – 100) |  | 94.6 (87.9 – 97.7) | 100 (97 – 100) |
| Rwanda | Roche cobas HCV plasma | 93.5 (86.6 – 97.0) | 97.2 (92.0 – 99.0) |  | 92.5 (85.3 – 96.3) | 100 (96.4 – 100) |  | 93.5 (86.6 – 97.0) | 99.1 (94.8 – 99.8) |

CI, confidence interval; DBS, dried blood spot; PSC, plasma separation card.
