# Supplementary material for: Sensitivity and specificity of Dried Blood Spot and Plasma Separation Card samples for Hepatitis C Virus RNA Testing
Source: PLOS Glob Public Health. 2026 Mar 11;6(3):e0006082. doi: 10.1371/journal.pgph.0006082 (PMC12978484; doi:10.1371/journal.pgph.0006082)
Supplement: S4 Table — DBS, dried blood spot; PSC, plasma separation card. (DOCX) [file pgph.0006082.s004.docx]

**S4 Table**. Proportion of invalid results according to assay, sample type, and site.

| **Assay and sample type** | **Georgia** | **Cameroon** | **Greece** | **Rwanda** | **All sites** |
| --- | --- | --- | --- | --- | --- |
| **Roche cobas 4800** |  |  |  |  |  |
| Venous DBS | 0/270 (0%) | 0/249 (0.00%) | 0/218 (0.00%) | 2/200 (1.0%) | 2/938 (0.21%) |
| Capillary DBS | 1/270 (0.37%) | 0/249 (0.00%) | 1/219 (0.46%) | 5/204 (2.45%) | 7/942 (0.74%) |
| Venous PSC | 3/270 (1.11%) | 3/252 (1.19%) | 2/220 (0.90%) | 1/200 (0.5%) | 10/942 (1.07%) |
| **Roche cobas 6800** |  |  |  |  |  |
| Venous DBS | 1/271 (0.37%) | 0/249 (0.00%) | 1/218 (0.46%) | 1/200 (0.5%) | 3/938 (0.32%) |
| Venous PSC | 0/270 (0.00%) | 2/251 (0.80%) | 0/218 (0.00%) | 1/200 (0.5%) | 3/939 (0.32%) |
| Capillary PSC | 2/270 (0.74%) | 1/249 (0.40%) | 1/218 (0.46%) | 1/199 (1.01%) | 6/936 (0.64%) |

DBS, dried blood spot; PSC, plasma separation card.
