# Supplementary figures and images for: Sensitivity and specificity of Dried Blood Spot and Plasma Separation Card samples for Hepatitis C Virus RNA Testing
Source: PLOS Glob Public Health. 2026 Mar 11;6(3):e0006082. doi: 10.1371/journal.pgph.0006082 (PMC12978484; doi:10.1371/journal.pgph.0006082)

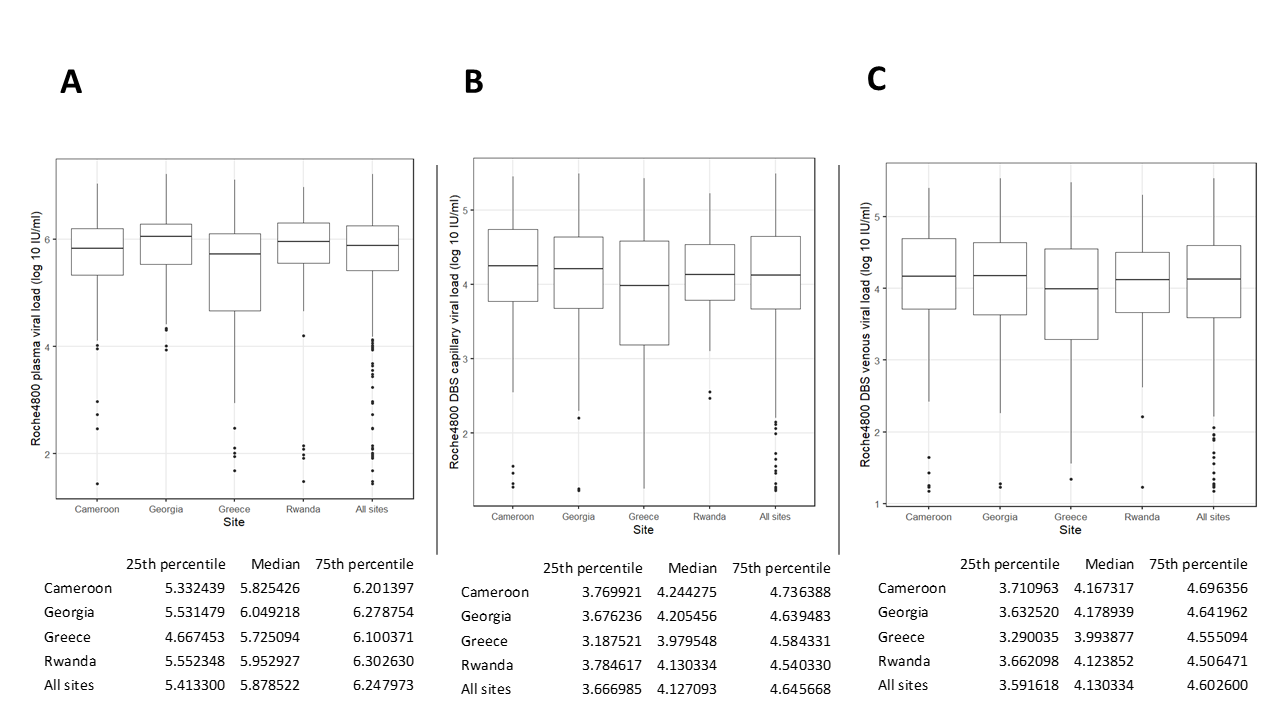

Supplement: S1 Fig — (A) Plasma; (B) capillary DBS; (C) venous DBS. The line in the middle of each box represents the median viral load; the top and bottom of each box represent the 75th and 25th centiles, respectively. DBS, dried blood spot. (TIF) [file pgph.0006082.s005.TIF]

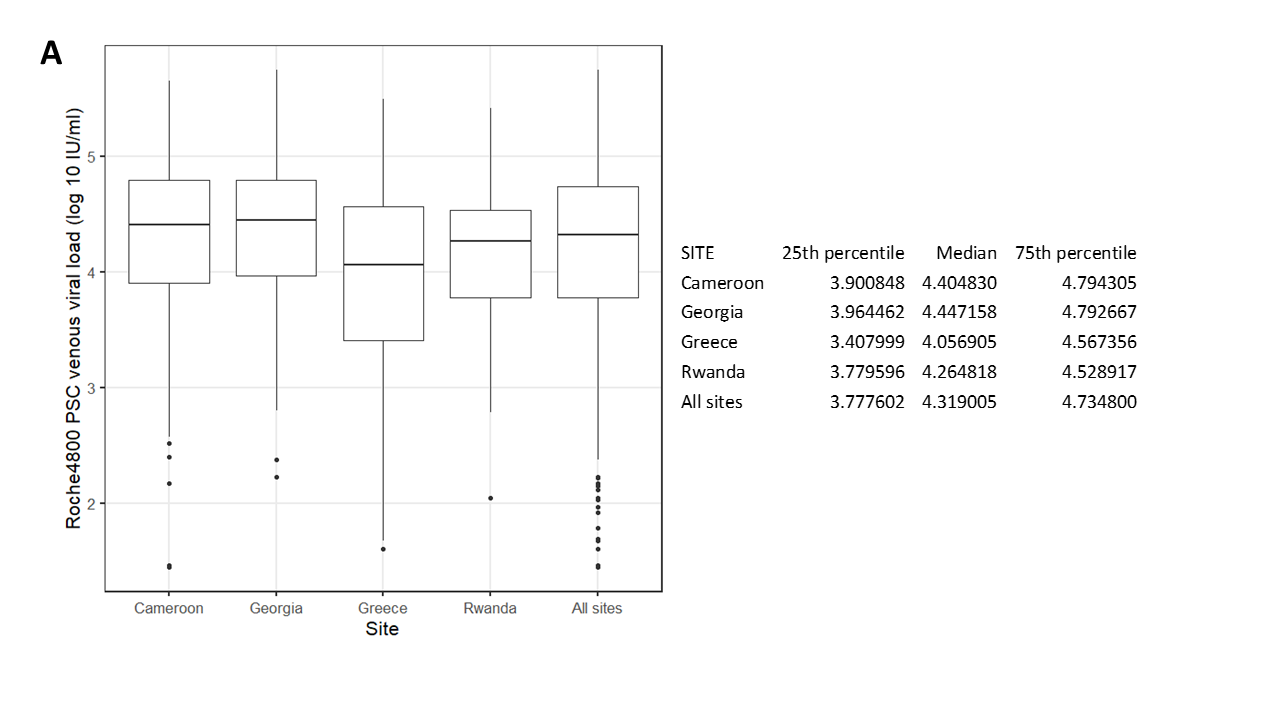

Supplement: S2 Fig — (A) venous PSC. The line in the middle of each box represents the median viral load; the top and bottom of each box represent the 75th and 25th centiles, respectively. PSC, plasma separation card; HCV, hepatitis C virus. (TIF) [file pgph.0006082.s006.TIF]

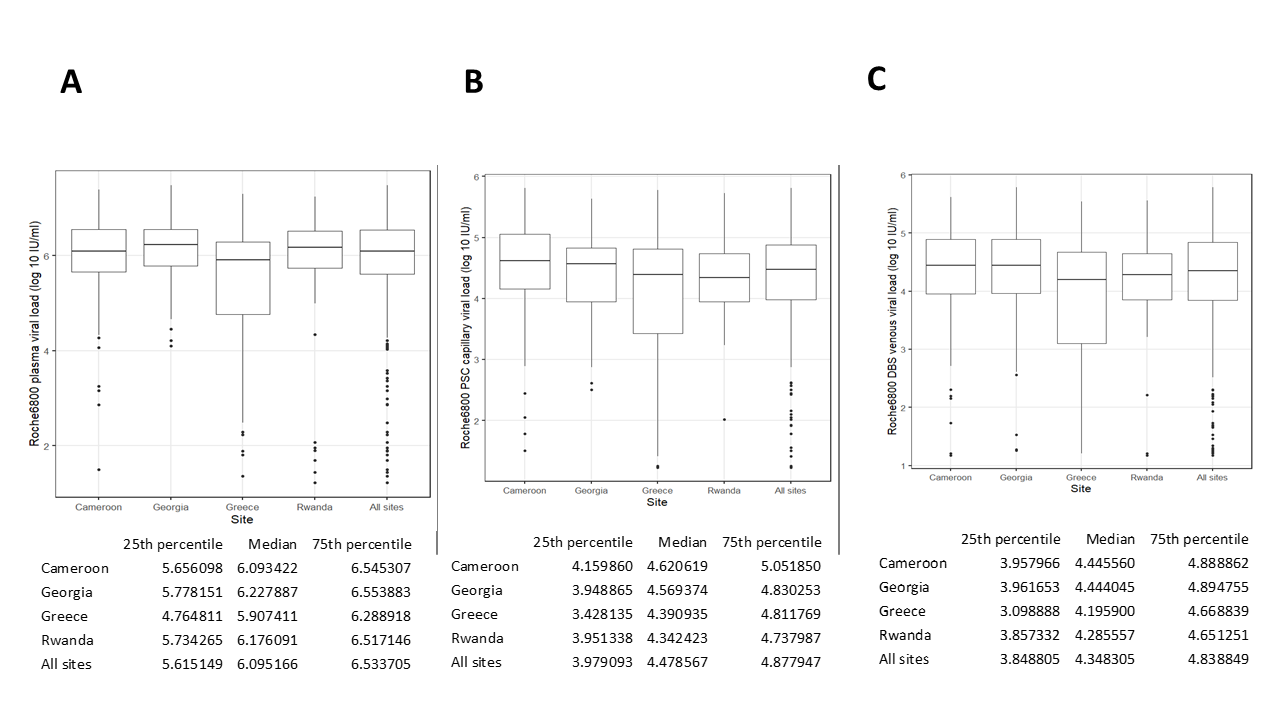

Supplement: S3 Fig — (A) Plasma; (B) capillary PSC; (C) venous DBS. The line in the middle of each box represents the median viral load; the top and bottom of each box represent the 75th and 25th centiles, respectively. DBS, dried blood spot; PSC, plasma separation card; HCV, hepatitis C virus. (TIF) [file pgph.0006082.s007.TIF]

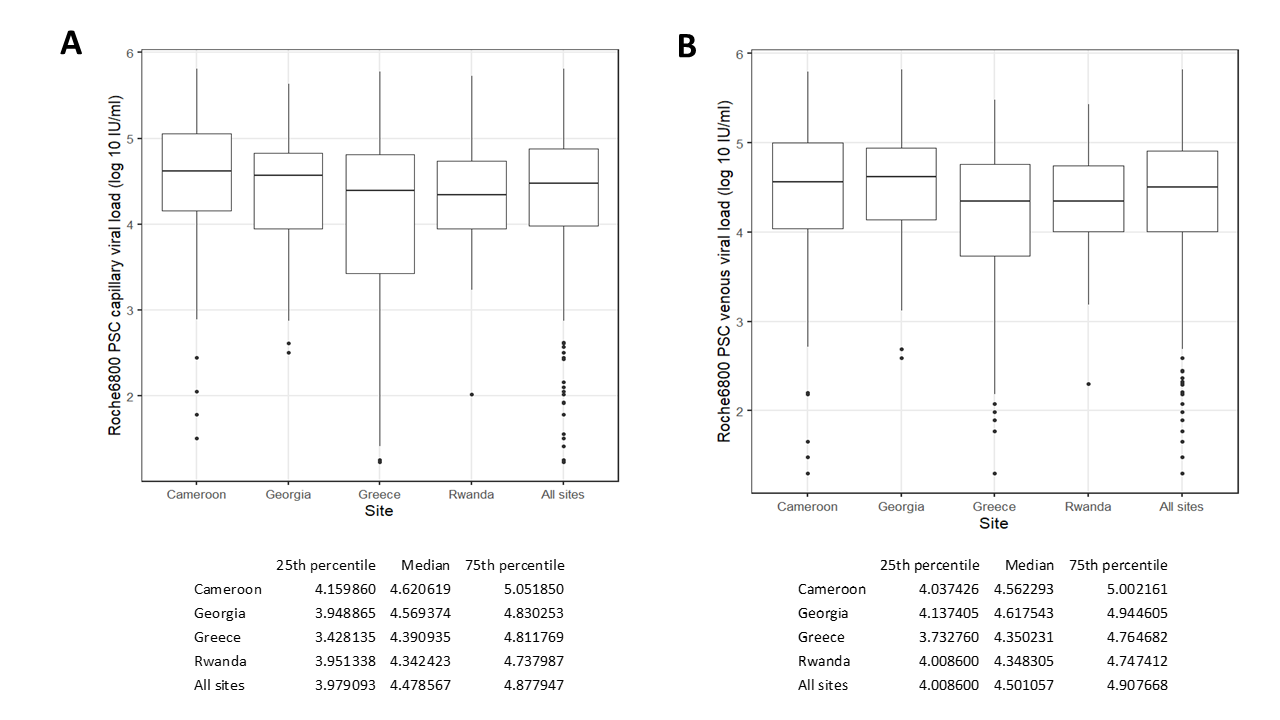

Supplement: S4 Fig — (A) capillary PSC; (B) venous PSC. The line in the middle of each box represents the median viral load; the top and bottom of each box represent the 75th and 25th centiles, respectively. Abbreviations: PSC, plasma separation card dried; HCV, hepatitis C virus. (TIF) [file pgph.0006082.s008.TIF]
