# Supplementary material for: Sensitivity and specificity of Dried Blood Spot and Plasma Separation Card samples for Hepatitis C Virus RNA Testing
Source: PLOS Glob Public Health. 2026 Mar 11;6(3):e0006082. doi: 10.1371/journal.pgph.0006082 (PMC12978484; doi:10.1371/journal.pgph.0006082)
Supplement: Attachment — Submitted filename: PLOS Global Health Issues Addressed_Feb 2026.pdf [file pgph.0006082.s010.pdf]

1. Please include a complete copy of PLOS' questionnaire on inclusivity in global research in your revised manuscript. Our policy for research in this area aims to improve transparency in the reporting of research performed outside of researchers' own country or community. The policy applies to researchers who have travelled to a different country to conduct research, research with Indigenous populations or their lands, and research on cultural artefacts. The questionnaire can also be requested at the journal's discretion for any other submissions, even if these conditions are not met. Please find more information on the policy and a link to download a blank copy of the questionnaire here: https://journals.plos.org/globalpublichealth/s/best-practices-in-research-reporting. Please upload a completed version of your questionnaire as Supporting Information when you resubmit your manuscript.

The questionnaire has been completed and uploaded as Supporting information.

2. Thank you for providing the following Financial Disclosure statement:

"This research was funded by UNITAID, grant number UA-HCV01. The funder had no role in study design, data collection and analysis, decision to publish, or preparation of the manuscript.

Roche Diagnostics provided the reagents for testing at the Australian site - National Reference Laboratory, Victoria, Australia.

FIND staff; EIR, FMJL, AM (Agnes Malobela), AM (Aurélien Mace), EF were funded through UNITAID, grant number UA-HCV01.

The funder had no role in study design, data collection and analysis, decision to publish, or preparation of the manuscript."

We note that "Roche Diagnostics" is a commercial funder (i.e. not an educational institution, non-profit, or government organization). Please note that it is required that authors disclose any commercial funding in their Competing Interests statement. Therefore, we ask that you please respond to the following queries:

a) Please confirm that there are no patents, products in development, or marketed products associated with this research to declare.

To the best of our search in publicly available literature and patent records, Roche currently markets HCV RNA detection assays for the cobas® 4800, 5800, 6800, and 8800 platforms. The cobas® Plasma Separation Card is a commercially available

*sample collection device that is associated with patents covering its design and use for plasma separation. The regulatory approval status of the Plasma Separation Card specifically for HCV RNA testing varies by region. We are not aware of any additional patented products****, in-development*** *diagnostics, or* ***separate marketed products*** *beyond these associated Roche assays and the plasma separation card in connection with HCV RNA detection.*

b) Please confirm that this funding does not alter your adherence to PLOS ONE policies on sharing data and materials.

We confirm that this funding and the above competing interests do not affect our adherence to PLOS ONE policies on the sharing of data and materials.

Using your responses to the queries above, we will update your Competing Interests statement on your behalf, or you may provide a drafted statement in your response. For more information on PLOS's guidelines regarding Competing Interests, please see here: https://journals.plos.org/globalpublichealth/s/competing-interests

2. We note that you have indicated that there are restrictions to data sharing for this study. For studies involving human research participant data or other sensitive data, we encourage authors to share de-identified or anonymized data. However, when data cannot be publicly shared for ethical reasons, we allow authors to make their data sets available upon request. For information on unacceptable data access restrictions, please see http://journals.plos.org/globalpublichealth/s/data-availability#loc-unacceptable-data-access-restrictions.

Before we proceed with your manuscript, please address the following prompts:

a) If there are ethical or legal restrictions on sharing a de-identified data set, please explain them in detail (e.g., data contain potentially identifying or sensitive patient information, data are owned by a third-party organization, etc.) and who has imposed them (e.g., a Research Ethics Committee or Institutional Review Board, etc.). Please also provide contact information for a data access committee, ethics committee, or other institutional body to which data requests may be sent.

There are no ethical or legal restrictions on sharing de-identified data sets. Where applicable, data-sharing agreements with participating countries were established.

b) If there are no restrictions, please upload the minimal anonymized data set necessary to replicate your study findings to a stable, public repository and provide us with the relevant URLs, DOIs, or accession numbers. Please see http://www.bmj.com/content/340/bmj.c181.long for guidelines on how to de-identify and prepare clinical data for publication. For a list of recommended repositories, please see https://journals.plos.org/globalpublichealth/s/recommended-repositories. You also have the option of uploading the data as Supporting Information files, but we would recommend depositing data directly to a data repository if possible.

The data sets have been uploaded on Figshare here:
https://figshare.com/articles/dataset/Roche_DBS_and_Plasma_Separation_Data_Set_-_HCV_RNA_Detection/29543879

Please update your Data Availability statement in the submission form accordingly.

The Data Availability statement has been updated accordingly.

The datasets analysed during the current study are available in the Figshare repository, Where applicable, data-sharing agreements with participating countries were established.
https://figshare.com/articles/dataset/Roche_DBS_and_Plasma_Separation_Data_Set_-_HCV_RNA_Detection/29543879

3. Please provide separate figure files in .tif or .eps format only and remove any figures embedded in your manuscript file. Please also ensure that all files are under our size limit of 10MB. Please leave the figure captions in the manuscript.

The figures embedded within the manuscript have been removed, and only the corresponding figure captions are provided at the end of the manuscript.

For more information about how to convert your figure files please see our guidelines: https://journals.plos.org/globalpublichealth/s/figures

4. Some material included in your submission may be copyrighted. According to PLOS's copyright policy, authors who use figures or other material (e.g., graphics, clipart, maps) from another author or copyright holder must demonstrate or obtain permission to publish this material under the Creative Commons Attribution 4.0 International (CC BY 4.0) License used by PLOS journals. Please closely review the details of PLOS's copyright requirements here: PLOS Licenses and Copyright. If you need to request

permissions from a copyright holder, you may use PLOS's Copyright Content Permission form.

Please respond directly to this email or email the journal office and provide any known details concerning your material's license terms and permissions required for reuse, even if you have not yet obtained copyright permissions or are unsure of your material's copyright compatibility.

Potential Copyright Issues:

“DBS_protocol_v1.0 9Apr2019_allsites.pdf” contains branding/a logo. We are not permitted to publish this under our CC-BY 4.0 license, even with permission. We ask that you please remove or replace it.

The DBS Protocol has been removed from the submission files.

Your manuscript has been returned to your account. Please log on to PLOS Editorial Manager at https://www.editorialmanager.com/pgph/ to access your manuscript.

Your manuscript can be found in the "Revisions Sent Back to the Author" link under the New Submissions menu. After you have made the changes requested above, please be sure to view and approve the revised PDF after rebuilding the PDF to complete the resubmission process.
